# Current use of modern contraceptives among young people 10 - 24 years in Central and Western Uganda: a cross-sectional study

**DOI:** 10.4314/ahs.v25i4.10

**Published:** 2025-12

**Authors:** Nelson Bunani, Raymond Tweheyo, Evelyne Baelvina Nyachwo, Allen Kabagenyi, Stella Neema, Elizeus Rutebemberwa

**Affiliations:** 1 Department of Health Policy, Planning and Management, Makerere University School of Public Health, Uganda; 2 Department of Population Studies, Makerere University School of Statistics and Planning, Uganda; 3 Department of Anthropology, Makerere University School of Social Sciences, Uganda

**Keywords:** Knowledge, modern contraceptive use, young people, Uganda

## Abstract

**Background:**

Despite being the most effective way to prevent unintended pregnancies and related complications, modern contraceptive use in Uganda remains low compared to the national target. We aimed to assess the knowledge and factors associated with the current use of modern contraceptive among young people aged 10-24 in central and western Uganda.

**Methods:**

This was a cross-sectional study that collected data from 289 in- and out-of-school young people aged 10-24 years in five districts of western and central Uganda. Modified Poisson regression was used to determine the factors associated with the current use of modern contraceptive use.

**Results:**

Overall, 62.6% (181) of the respondents knew about modern contraceptives. Current use of modern contraceptives was 58.5% (169). Current use of modern contraceptives was associated with being aged 20-24 (Adj. PRR= 0.69, 95%CI; 0.52-0.90), p=0.007; not residing with someone as a sexual partner (Adj. PRR=0.71, 95%CI; 0.57-0.88), p=0.002; and being a student (Adj. PRR=1.37, 95% CI; 1.04-1.79), p=0.023.

**Conclusion:**

Young people aged 10-24 in central and western Uganda were well-informed about modern contraceptives, especially injectables and condoms, but less knowledgeable about emergency contraceptives. Students used modern contraceptives more than non-students, while those aged 20-24 and not cohabiting with partners were less likely to use them. Awareness campaigns for 20-24-year-olds and those living with partners are necessary to increase usage.

## Background

Globally, an estimated 21 million young girls aged 15-19 years become pregnant every year with the majority of the pregnancies unintended^1^. Additionally, an estimated 2.5 million girls in developing countries gave birth by age 16 in the year 2018^2^, most births were due to unintended pregnancies. The highest burden of teenage pregnancies is found in low and middle-income countries, particularly in sub-Saharan Africa (SSA) and the Caribbean^2^. In 2013, SSA had the highest burden of teenage pregnancies globally^3^, which has since then been high, with 21.5% of teenage pregnancies in East Africa^4^, and in Uganda, the teenage pregnancy rate is at 25%^5^.

Early age pregnancy (before 18 years) affects young people's sexual and reproductive health^6^. Additionally, the reproductive choices made by young people greatly impact their health, education, future aspirations and their transition into adulthood^7^. Available evidence reveals that early pregnancy and childbearing contributors include individual level, community and health system-related factors^8^-^10^.

Although it is most effective for averting unintended pregnancies and the related adverse reproductive events for the mother and baby^11^, modern contraceptive use in Uganda remains low, estimated at 35%, versus 50% national target for the year 2020^12^. Low use of modern contraceptives could partly explain the persistently high total fertility rate (TFR) in Uganda which has slowly declined from 7.4 in 1988-1989 to 5.4 children per woman in 2016^5^. Teenage pregnancy is a key contributor to high fertility rates and risky pregnancies, which have resulted in the persistently high maternal mortality rate in Uganda^5^,^13^.

While the government of Uganda made a commitment to improve the quality of life and well-being of young people^14^, this population segment–especially the adolescents and youth are a key inaccessible population for modern contraceptives. Central and western Uganda have registered a high TFR in the past years compared to other areas^15^. This study aimed at determining the knowledge of, and factors associated with current use of modern contraceptives among young people 10-24 years in central and western Uganda.

## Methods

### Study design

This was a cross-sectional study. A semi-structured questionnaire designed in CSPro version 7.5.1 was used to collect the data from young people aged 10-24 years. Standardized modules of the Demographic and Health Survey (DHS) questionnaire on sexual and reproductive health for knowledge on family planning were adapted for this study.

### Study setting

The study was conducted in the five districts of western and central Uganda. They included; Gomba, Kyegegwa, Kibaale,, Bundibugyo and Buliisa. These districts were selected because they have a higher population growth rate than the national average of 3%, and by 2020 these districts had a total of 2,041,106 people representing about 5% of Uganda's total population^16^. The TFR in these five districts ranges from 4.7 in Gomba to 7.8 children per woman per annum in Kyegegwa^16^. Additionally, a study conducted in Uganda identified these districts as fertility hot spots^17^. We conducted this study during the COVID-19 lockdown restrictions when all the schools were closed, so the participants were located in their communities. We conducted this study between July and August 2020.

### Sampling and sample size

A sample of 289 respondents was computed using Kish Leslie formula for cross sectional studies^18^. This number was proportionately divided across the different study districts. Simple random sampling using computer generated random numbers was used to select the study participants. In the communities, house-to-house assessments were done to find eligible young people who were then classified as either in-school or out-of-school. Respondents were asked the last time they were in school. Those who were in school by March 2020 (when the COVID-19 lockdown restrictions were instituted) were considered as being in school. At house hold level, only one respondent who met the inclusion criteria was interviewed.

### Study variables

The primary outcomes for this study were knowledge of modern contraceptives and current use of modern contraceptive. Knowledge of modern contraceptive was measured using 9 questions that were used to assess respondent's knowledge of modern contraceptives. Each of the questions measuring knowledge had a “Yes” and “No” responses where a “Yes” was considered to be a correct response and a “No” an incorrect response.response. A respondent was considered to have good knowledge if he scored more than 6 out of the 9 knowledge questions. Respondents who scored less that 6 out of the 9 knowledge questions were considered to have low knowledge^19^. Current use of modern contraceptives was recorded as “Yes” and “No”. Respondents currently using any modern contraceptive were recoded as “Yes” and those who were not using were recorded as “No”. Current use of modern contraceptives was defined as a respondent currently using any modern contraceptive method during sexual intercourse. The modern contraceptives considered in this study were; emergency pills, Intrauterine devices (IUDs), male condoms, injectable contraceptives and Implants. Independent variables were; sex of the respondent, age at first sexual intercourse, marital status, education level, occupation, religion, ever heard of any family planning (FP) method, access to FP service, distance to the health facility and the district of origin.

### Data management and analysis

A digital questionnaire designed in CSPro 7.5.1 was used as an interviewer-administered tool from computer tablets (personal digital assistants). The questionnaire was translated into the appropriate local language for the region: Luganda, Runyoro and Rutooro. Questionnaires included socio-demographic variables, questions on knowledge of modern contraceptives, health system related questions on access to family planning services, and assessments for risky behaviors, such as unprotected sexual intercourse, alcohol and other substance use. Six research assistants (RAs) with a minimum of a bachelor's degree and fluent in the appropriate language were trained on research ethics, field conduct and how to administer the questionnaire from the personal digital assistant (PDA) and sync data to the server. Daily editing of data was done, reviewed by a team leader, and data were synced to a central server at the Uganda Bureau of Statistics. Completed datasets were downloaded into Microsoft excel, and corresponding (CSpro files) cleaned before transfer to STATA software for analysis. Datasets held de-identified data, and alongside the computer folders were encrypted for data security.

Univariate, Bivariable, and multivariable analyses were conducted in STATA SE version 15 to explore the data, cross-tabulate the dependent and independent variables, and generate inferential statistics. We further performed stratified analysis to determine the level of current use of modern contraceptives by socio-demographic characteristics across the study districts. To determine the association between independent variables and current use of modern contraceptives, we used a generalized linear model (glm) using modified Poisson regression with robust variances at bivariable and multivariable analysis^20^. Independent variables significant at p<0.2 in the bivariable cross tabulations, and all plausible variables, were included in the final multivariable model. Then model building was carried out using stepwise elimination method to obtain the variables that were associated with current use of modern contraceptives, prevalence rate ratios (PRRs) with the corresponding 95% confidence intervals were obtained and presented in a table.

## Results

### Current use of modern contraceptive use by selected background characteristics across the study districts

Out of the 289 study respondents, 76 (27%) were in schools. More than half of the respondents, 184 (63.7%) were females. The mean age of the respondents was 20.8 ±2.4 years, and at least half were aged 17 at sexual debut, 146 (50.5 %). Furthermore, most respondents, 278 (96.2%), had never heard about family FP methods. (Table 1).

**Table 1:** Back ground characteristics of the respondents

Variables	(N=289, %)
**Sex**	
Female	184 (63.7)
Male	105 (36.3)
**Age of the respondents**	
≤16	14 (4.8)
17-19	77 (26.6)
20-24	198 (68.5)
**Age at first sexual intercourse**	
≤14	51 (17.7)
15-17	146 (50.5)
17-24	92 (31.8)
**Marital status**	
Married	121 (41.9)
Not married	168 (58.1)
**Reside with someone as a sexual partner**	
Yes	121 (41.9)
No	168 (58.1)
**Education level**	
No education	14 (4.8)
Primary	131 (45.3)
Secondary	126 (43.6)
Tertiary	18 (6.2)
**Occupation**	
Agriculture worker	101 (35.9)
Salaried employment	16 (5.7)
Business	88 (31.3)
Student	76 (27.1)
**Region**	
Central	50 (17.3)
Western	239 (82.7)
**Religion**	
Catholics	145 (50.2)
Protestants	77 (26.6)
Muslims	25 (8.7)
Others*	42 (14.5)
**Ever heard of any FP method**	
Yes	278 (96.2)
No	11 (3.8)
**Access to FP services**	
Yes	181 (62.6)
No	108 (37.4)
Distance to the nearest Health facility**	
≤1Km	177 (68.3)
2-4Kms	54 (20.9)
≥5Kms	28 (10.8)

* Other religions included; Pentecostal and seventh day Adventists

** variable with missing data

### Current use of modern contraceptive use by selected background characteristics across the study districts

Overall, 58.5% (169) of the respondents were currently using modern contraceptives, and at least half of the respondents with a secondary level of education in the districts of Bundibugyo, Gomba, Kibaale, and Kyegegwa were currently using modern contraceptives. Kyegegwa district had a higher number of respondents, 21(87.5%) that were currently using modern contraceptives. (Table 2).

**Table 2:** Prevalence of modern contraceptive use by selected background characteristics across the districts

Variable	District									
	Buliisa (n=68)		Bundibugyo(n=63)		Gomba (n=50)		Kibaale (n=56)		Kyegegwa (n=52)	
	Yes (n, %)	No (n, %)	Yes (n, %)	No (n, %)	Yes (n, %)	No (n, %)	Yes (n, %)	No (n, %)	Yes (n, %)	No (n, %)
**Education level**										
No education	9(23.7)	2(6.7)	0(0.0)	1(6.7)	0(0.0)	0(0.0)	0(0.0)	0(0.0)	0(0.0)	2(7.1)
Primary	22(57.9)	23(76.7)	21(43.8)	5(33.3)	12(40.0)	13(65.0)	10(34.5)	5(18.5)	8(33.3)	12(42.9)
Secondary	5(13.2)	5(16.7)	25(52.1)	9(60.0)	17(56.7)	5(25.0)	17(58.6)	16(59.3)	13(54.2)	14(50.0)
Tertiary	2(5.3)	9(0.0)	2(4.2)	0(0.0)	1(3.3)	2(10.0)	2(6.9)	6(22.2)	3(12.5)	0(0.0)
**Marita status**										
Married	26(68.4)	12(31.6)	12(25.0)	36(75.0)	9(30.0)	21(70.0)	6(20.7)	23(79.3)	7(29.2)	17(70.8)
Not married	20(66.7)	10(33.3)	7(46.7)	8(53.3)	10(50.0)	10(50.0)	8(29.6)	19(70.4)	16 (57.1)	12(42.9)
**Age of the respondents**										
≤16	1(2.6)	4(13.3)	1(2.1)	3(20.0)	0(0.0)	2(10.0)	0(0.0	0(0.0)	1(4.2)	2(7.1)
17-19	11(29.0)	6(20.0)	15(31.3)	3(20.0)	10(33.3)	10(50.0)	6(20.7)	7(25.9)	2(8.3)	7(25.0)
20-24	26(68.4)	20(66.7)	32(66.7)	9(60.0)	20(66.7)	8(40.0)	23(79.3)	20(74.1)	21(87.5)	19(67.9)
**Age at first sexual intercourse**										
≤14	11(29.0)	9(30.0)	14(29.2)	2(13.3)	5(16.7)	2(10.0)	1(3.5)	3(11.1)	1(4.2)	3(10.7)
15-17	19(50.0)	17(56.7)	23(47.9)	10(66.7)	14(46.7)	11(55.0)	16(55.2)	15(55.6)	8(33.3)	13(46.4)
17-24	8(21.1)	4(13.3	11(22.9)	3(20.0)	11(36.7)	7(35.0)	12(41.4)	9(33.3)	15(62.5)	12(42.9)

### Knowledge of modern contraceptives among young people aged 10-24 years

As depicted in Table 3, out of the 289 respondents, 181(62.6%) had high knowledge of modern contraceptives. Most respondents were highly knowledgeable on injectable contraceptives 278 (96.2%) and male condoms 279 (96.5%). There was low knowledge of modern contraceptives, 138 (47.8%).

**Table 3:** Knowledge of modern contraceptives among young people aged 10-24 years

Variables	Frequency (n=289)	Percent (%)
Women can have an operation to avoid having any more children (female sterilization)	210	72.7
Men can have an operation to avoid having any more children (male sterilization)	181	62.6
Women can have a loop or coil placed inside them by a doctor or a nurse, which can prevent them from having a p regnancy for one or more years (IUDs)	217	75.1
Women can have an injection by a health provider that prevents them from becoming pregnant for one or more years (injectable)	278	96.2
Women can have one or more rods placed in their upper arm by a doctor or nurse that can prevent pregnancy for one or more years (implant)	247	85.5
Women can take a pill every day to avoid becoming pregnant (pill)	249	86.2
Men can put a rubber sheath on their penis before sexual intercourse to prevent becoming pregnant (Male condom)	279	96.5
Women can place a sheath in their vagina before sexual intercourse to prevent pregnancy (Female condom)	172	59.5
As an emergency measure, within three days of having un protected sexual intercourse, women can take an emergency pill to prevent pregnancy (Emergency contraception)	138	47.8
Overall knowledge	181	62.6

### Current use of modern contraceptives by district

**Figure 1:**
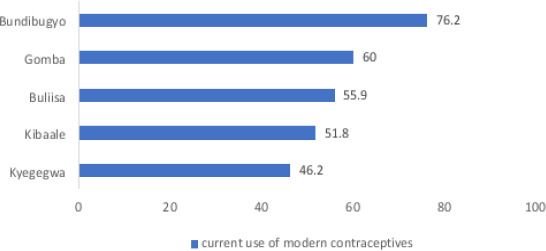
Modern contraceptive use by district

Bundibugyo district had the highest number (76.2%) of respondents who were currently using modern contraceptives. (Figure 1)

### Factors associated with the current use of modern contraceptives among young people 10 – 24 years in central and western Uganda

At bivariable analysis, being aged above 16 years, marital status, being a student and having access to FP from a health facility appeared significantly associated with modern contraceptive use. At multivariable analysis, age, being married, and being a student were significantly associated with the current use of modern contraceptives.

The prevalence of current use of modern contraceptives was 31% times lower among the respondents aged 20-24 years compared to those who were aged 16 years and below (Adj. PRR= 0.69, 95%CI; 0.52-0.90), p=0.007. Additionally, the prevalence of current use of modern FP was 29% times lower among the respondents who were not married compared to those who resided with someone as sexual partner (Adj. PRR=0.71, 95%CI; 0.57-0.88), p=0.002. The prevalence of current use of modern FP was 37% times higher among the respondents that were students compared to those who had no formal education (APR=1.37, 95% CI; 1.04-1.79), p=0.023. (Table 4).

**Table 4:** Factors associated with the current use of modern contraceptives among young people aged 10-24 years

variables	Current use of modern contraceptives		Crude PRR (95%CI)	P-value	Adj.PRR (95% CI)	P-value
	**Yes (n, %)**	**No (n, %)**				
**Sex**						
Female	100 (54.4)	84 (45.7)	1		1	
Male	69 (65.7)	36 (34.3)	0.80 (0.63-1.01)	0.065	0.96 (0.76-1.20)	0.715
**Age**						
≤16	3 (21.4)	11 (78.6)	1		1	
17-19	44 (57.1)	33 (42.9)	0.60 (0.43-0.82)	0.002	0.78 (0.58-1.05)	0.108
20-24	122 (61.6)	76 (38.4)	0.55 (0.42-0.73)	<0.001	0.69 (0.52-0.90)	0.007**
**Age at first sexual intercourse**						
≤14	32 (62.8)	19 (37.3)	1			
15-17	80 (54.8)	66 (45.2)	1.16 (0.85-1.59)	0.336		
17-24	57 (62.0)	35 (38.0)	1.02 (0.72-1.43)	0.926		
**Marital status**						
Married	60 (35.3)	109 (64.5)	1		1	
Not married	61 (50.8)	59 (49.2)	0.75 (0.61-0.93)	0.009	0.71 (0.57-0.88)	0.002**
**Education level**						
No education	9 (64.3)	5 (35.7)	1			
Primary	73 (55.7)	58 (44.3)	1.18 (0.68-2.07)	0.556		
Secondary	77 (61.1)	49 (38.9)	1.07 (0.61-1.88)	0.819		
Tertiary	10 (55.6)	8 (44.4)	1.19 (0.60-2.34)	0.621		
**Occupation**						
Agriculture worker	67 (66.3)	34 (33.7)	1		1	
Salaried employment	9 (56.3)	7 (43.8)	1.22 (0.75-2.0)	0.416	1.37 (0.83-2.26)	0.217
Business	55 (62.5)	33 (37.5)	1.09 (0.81—1.46)	0.583	1.11 (0.86-1.44)	0.418
Student	36 (47.4)	40 (52.6)	1.42 (1.09-1.87)	0.011	1.37 (1.04-1.79)	0.023*
**Region**						
Central	30 (60.0)	20 (40.0)	1			
Western	139 (58.2)	100 (41.8)	1.04 (0.77-1.39)	0.812		
**Religion**						
Catholics	83 (57.2)	62 (42.8)	1			
Protestants	48 (62.3)	29 (37.7)	0.91 (0.69-1.18)	0.467		
Muslims	16 (64.0)	9 (36.0)	0.87 (0.57-1.34)	0.539		
Others*	22 (52.4)	20 (47.6)	1.09 (0.81-1.47)	0.570		
**Ever heard of any FP method**						
Yes	164 (59.0)	114 (41.0)	1			
No	5 (45.5)	6 (54.6)	1.26 (0.79-2.0)	0.328		
**Distance to the nearest Health facility**						
≤1Km	104 (58.8)	73 (41.2)	1			
2-4Kms	34 (63.0)	20 (37.0)	0.92 (0.68-1.24)	0.587		
≥5Kms	16 (57.1)	12 (42.9)	1.03 (0.71-1.49)	0.871		
**Knowledge**						
Low knowledge	57 (52.8)	51 (47.2)	1		1	
High Knowledge	112 (61.9)	69 (38.1)	0.84 (0.68-1.05)	0.125	1.00(0.81-1.24)	0.980

PRR-prevalence rate ratio, Adj.PRR-Adjusted prevalence rate ratio

* p<0.05

** P<0.01

** P<0.001

## Discussion

Modern contraceptive knowledge was found to be 62.6%. The most commonly known modern contraceptives were male condoms, injectables and implants. The reported level of knowledge is higher than that reported in the UDHS^21^. The higher level of knowledge may be due to increased access to information regarding family planning through radio and television^22^. It is also higher than that reported in Ethiopia at 52.1 %^23^, but lower than that found in the general population in Gambia, where 89.4% of the population knew about progesterone only and contraceptives^24^,^25^,^26^.

Our study further found the current use of modern contraceptive to be 58.5% among young people, which was higher than that previously reported among adolescents in Uganda at 30.9%^27^. The higher prevalence of modern contraceptive use in this study is likely due to increased knowledge^28^. The reported prevalence is higher than that reported in Mali at 17%^29^. The low use of modern contraceptives in Mali suggests that their use for fertility prevention is low, which may be due to limited awareness.

We also found that young people who were aged 20-24 years were less likely to use modern contraceptives. Low use of modern contraceptives could be attributed to the fact that people aged above 20 years may be possibly married or preparing to have children and, therefore, could not use contraceptives. Similarly, the UDHS 2016 reported low use of modern contraceptives among young women below 24 years compared to their older counterparts^5^. Contrary to our findings, a previous study in Uganda found a positive association between modern contraceptive use and being aged 24 or less^30^. Further, it has been stressed that youths and adolescents who are recently married are confronted with pressure to bear children, which makes them not use contraception^31^. This pressure may be from the community and close family members, such as parents who want grandchildren.

This study also found that young people who were not married were less likely to use modern contraceptives compared to those who co-resided with someone as a sexual partner. Most of the young people who were not residing with a sexual partner could not have been sexually active and, therefore, did not find any reason to use contraceptives. Our findings agree with those from a previous study in Ghana, which found that cohabiting and staying with someone as a sexual partner was associated with increased use of modern contraceptives^32^. Additionally, adolescents who reside with someone as a sexual partner could be using modern contraceptives because they have partner support. Partner support has shown a positive relationship with modern contraceptive use among adolescents in northwestern Tanzania^33^.

We also found that being a student was associated with the current use of modern contraceptives. This is attributed to the exposure to contraceptive information through media and the internet, which increases their knowledge of the benefits of contraceptives^34^, while others may use contraceptives due to fear of getting pregnant while in school, which ultimately increases contraceptive use. Comparable findings were reported by a study conducted in 20 African countries, which reported an increased contraceptive use and being in school^35^. Additionally, in northern Tanzania, being in school was associated with modern contraceptive use due to increased literacy levels that allow young people to access and understand family planning information easily^33^.

### Strength and limitations

We collected data during the COVID-19 lockdown restrictions and could have registered some young people as out of school, yet they were in school, which may have affected the findings. We verified this by asking them whether they were in school by the time of lockdown.

This cross-sectional study design cannot infer causality among variables under study but can only help formulate a hypothesis for further investigations using stronger study designs that can infer temporal relationships.

## Conclusion and implications

Young people 10- 24 years old in central and western Uganda were found highly knowledgeable of modern contraceptives, particularly injectables and condoms, but least knowledgeable of emergency contraception. Respondents who were students compared to non-students were more likely to be using modern contraceptives, while those aged 20-24 years and those not staying with their sexual partners were less likely to use modern contraceptives. It appears that persons aged 10 – 24 years are planning their marital life and have less likelihood of contraceptive use.

## Data Availability

To protect the confidentiality of participant information, ethical restrictions have been imposed on the data used in this study. Interested researchers may submit queries related to data access to MakSS REC or to the corresponding author.

## List of abbreviations

WHO: World Health Organization
UNFPA: United Nations Population Fund
REC: Research and Ethics Committee
UBOS: Uganda Bureau of Statistics
MOH: Ministry of Health
